# Network-based logistic regression integration method for biomarker identification

**DOI:** 10.1186/s12918-018-0657-8

**Published:** 2018-12-31

**Authors:** Ke Zhang, Wei Geng, Shuqin Zhang

**Affiliations:** 1 School of Mathematical Sciences, Fudan University, No.220 Handan Road, Shanghai, 200433 China; 2 Center for Computational Systems Biology, Shanghai Key Laboratory for Contemporary Applied Mathematics, School of Mathematical Sciences, Fudan University, No.220 Handan Road, Shanghai, 200433 China

**Keywords:** Data integration, Logistic regression, Meta-analysis, Network penalty

## Abstract

**Background:**

Many mathematical and statistical models and algorithms have been proposed to do biomarker identification in recent years. However, the biomarkers inferred from different datasets suffer a lack of reproducibilities due to the heterogeneity of the data generated from different platforms or laboratories. This motivates us to develop robust biomarker identification methods by integrating multiple datasets.

**Methods:**

In this paper, we developed an integrative method for classification based on logistic regression. Different constant terms are set in the logistic regression model to measure the heterogeneity of the samples. By minimizing the differences of the constant terms within the same dataset, both the homogeneity within the same dataset and the heterogeneity in multiple datasets can be kept. The model is formulated as an optimization problem with a network penalty measuring the differences of the constant terms. The *L*_1_ penalty, elastic penalty and network related penalties are added to the objective function for the biomarker discovery purpose. Algorithms based on proximal Newton method are proposed to solve the optimization problem.

**Results:**

We first applied the proposed method to the simulated datasets. Both the AUC of the prediction and the biomarker identification accuracy are improved. We then applied the method to two breast cancer gene expression datasets. By integrating both datasets, the prediction AUC is improved over directly merging the datasets and MetaLasso. And it’s comparable to the best AUC when doing biomarker identification in an individual dataset. The identified biomarkers using network related penalty for variables were further analyzed. Meaningful subnetworks enriched by breast cancer were identified.

**Conclusion:**

A network-based integrative logistic regression model is proposed in the paper. It improves both the prediction and biomarker identification accuracy.

**Electronic supplementary material:**

The online version of this article (10.1186/s12918-018-0657-8) contains supplementary material, which is available to authorized users.

## Background

Biomarker plays an important role in early detection, diagnosis, monitoring, and prevention of disease, and it also helps in evaluation of the safety and efficacy of new drugs or new therapies. With the fast development of biotechnologies, more and more biological data are available, such as the gene expression, miRNA expression, DNA methylation and so on (NCBI GEO [1], TCGA). This makes it much easier to identify the genes, proteins, miRNAs etc. as the biomarkers.

In recent years, many statistical models and computational algorithms have been developed to do variable selection, which can be applied to identify the biomarkers in both regression and classification problems [2–8]. A pioneering work in this area is LASSO [2]. It adds *L*_1_ penalty to the original least square problem, which leads to the sparsity of the coefficients and thus achieves the variable selection goal. Based on this idea, several other variable selection methods are proposed, such as the sparse logistic regression [9], sparse partial least square regression [4], sparse partial least square classification [3]. Due to the high co-linearity of some covariates, these methods may select different variables that have similar effects on the responses. To take into account this issue, elastic net model adds both the *L*_1_ and *L*_2_ norm as the penalty of the coefficients [10]. With a balance of both norms, the highly correlated variables can be selected together. This model is further extended to the network constraints with sparsity [11–14]. In [11], the *L*_2_ norm in elastic net model is changed to a Laplacian term, which penalizes the variables that have connections in a given network, such that the coefficients of these variables tend to be the same. In [12, 13], the coefficients in the Laplacian term are replaced by their absolute value, which considers the case that the highly correlated variables have opposite contributions to the response. Different computational algorithms are given in these two papers. By adding the network constraints, the variables having high correlations or connections can be selected together, which reduces the effect of co-linearity, and thus improves the variable selection robustness.

Though such a lot of methods have been developed for variable selection, these models are mainly for one single dataset. Due to the small sample size relative to the large number of variables and the batch effects in different platforms or different laboratories, the biomarkers inferred from one dataset often suffer a lack of reproducibilities. As a potential solution to such problems, integrative analysis is a cost-effective option, since many genomic databases are nowadays publicly available. For example, the public functional genomics data repository NCBI GEO has more than 2.5 billion samples on more than 18 thousand platforms [1]. Here, integrative analysis means combining the data or information from multiple independent studies that are designed for the same biological or medical problems in order to draw more reliable conclusions, though some integration methods focusing on incorporating different data types have been developed [15–23]. To this purpose, there mainly exist two types of approaches: analysis by data merging and meta-analysis. The merging approach integrates the same data type after transforming the original data to numerically comparable measures or correcting the confounder factors first [24–28], while the meta-analysis approach combines the results of individual studies at the interpretative level [29–37]. In the data merging approach, the first step is to do cross-platform normalization or confounder correction, followed by the variable selection methods for one single dataset. Compared to data merging approach, meta-analysis is more complex and has taken into account more factors in the integrative process. The key issues for guiding conducting a meta-analysis of gene expression microarray datasets has given in [30]. An early R package for implementing meta-analysis is in [29], which has been widely applied such as the work in [34, 37]. Later on, several methods were proposed on meta-analysis. Ma et al. first proposed a Meta Threshold Gradient Descent Regularization (MTGDR) approach [31], then they developed a 2-norm group bridge penalization approach such that the markers with consistent effects across multiple studies can be identified [32]. They further proposed a sparse boosting for marker identification [33]. Li et al. proposed meta-lasso (MetaLasso) method for variable selection in meta-analysis, which used a hierarchical decomposition on regression coefficients to identify important genes, and kept the selection flexibility across different datasets [36]. These methods are all based on logistic regression for selecting the genes in microarray datasets, without considering the gene-gene interactions or the high correlations between the genes. Though the work [35] presented a statistical framework for identifying the differential co-expressed gene pairs as markers, they did not consider the general gene-gene interactions.

In this article, we investigate the integrative analysis of multiple datasets from different platforms or laboratories that are designed for the same biological questions. We propose a penalization approach based on logistic regression for biomarker selection. The penalization includes two parts: penalization of the sample relations and penalization of the variables. Penalization of the sample relations defines a new penalty as the the function of the sample relation network, and aims to make the regression coefficients for the samples from the same source be the same while allowing the heterogeneity across different datasets. The advantages of taking into account the sample relation network in general regression have been addressed in [38]. The penalization of the variables takes advantage of the recent development on network constraints penalization methods in single dataset such that the variables having high correlation or given connections can be selected together, which cannot be easily extended to from the current integrative models [31–33, 36, 37]. Numerical experiments on both simulated datasets and real datasets show the performance of our formulation.

## Methods

We assume the variables are measured in *M* different experiments with *M*>1. Let *X*^*m*^ denote the measurement of the variables in the *m*-th experiment, which is an *N*^*m*^×*p* matrix with *N*^*m*^ being the sample size and *p* being the number of variables. We let $X^{m}_{i}$ denote the *i*-th row in *X*^*m*^. Let *Y*^*m*^ denote the clinical outcomes in the *m*-th experiment, which is a vector of binary values representing case/control state or different disease states, and $Y^{m}_{i}$ be the *i*-th entry of *Y*^*m*^. We let **X**=((*X*^1^)^*T*^,(*X*^2^)^*T*^,⋯,(*X*^*M*^)^*T*^)^*T*^ be the values of the variables for all the samples, and **Y**=((*Y*^1^)^*T*^,(*Y*^2^)^*T*^,⋯,(*Y*^*M*^)^*T*^)^*T*^ be the clinical outcomes for all the samples. Let **X**_*i*_ denote the *i*-th row in **X**, and **Y**_*i*_ the *i*-th entry in **Y**, correspondingly. $N=\sum _{m=1}^{M} N^{m}$ is the total number of samples for all the considered datasets.

We first consider the logistic regression model for each single dataset. Let $p_{i}^{m}=P\left (Y^{m}_{i}=1|X^{m}_{i}\right)$ denote the probability that the sample *i* in the *m*-th experiment has the outcome 1. Then the logistic regression model can be formulated as:

$$\log\left(\frac{p_{i}^{m}}{1-p_{i}^{m}}\right)=\beta^{m}_{0}+{X^{m}_{i}}\beta^{m}, i=1,2,\cdots,N^{m},$$

where $\beta ^{m}=\left (\beta ^{m}_{1},\beta ^{m}_{2},\cdots,\beta ^{m}_{p}\right)^{T}$. To obtain $\beta ^{m}_{0}, \beta ^{m}$, we can maximize the log-likelihood function, which can be formulated as a minimization problem as follows:

1 $$\begin{array}{@{}rcl@{}} \min_{\beta_{0}^{m},\beta^{m}}\,\, -{\ell} \left(\beta_{0}^{m},\beta^{m}\right), \end{array} $$

where ${\ell } \left (\beta _{0}^{m},\beta ^{m}\right)=\sum _{i=1}^{N^{m}} \left (Y^{m}_{i}\cdot \left (\beta _{0}^{m}+X_{i}^{m}\beta ^{m}\right)\right.-\log \left (1+\exp \left (\beta _{0}^{m}+X_{i}^{m}\beta ^{m}\right)\right).$

To do biomarker identification using logistic regression model, different penalties have been proposed to add to (1), which can be formulated as:

2 $$\begin{array}{@{}rcl@{}} \min_{\beta_{0}^{m},\beta^{m}}\,\, -\frac{1}{N^{m}}\ell{\left(\beta_{0}^{m},\beta^{m}\right)}+\lambda P_{\alpha}\left(\beta^{m}\right), \lambda>0, \end{array} $$

where *λ* is a parameter to control the importance of the regularization term.

One formulation of *P*_*α*_(*β*^*m*^) is $P_{\alpha }(\beta ^{m})=\frac {1}{2}(1-\alpha) \|\beta ^{m}\|_{2}^{2}+\alpha \|\beta ^{m}\|_{1}$. When *α*=0, it corresponds to ridge penalty, when *α*=1, it corresponds to the LASSO [2], and when 0<*α*<1, it corresponds to the elastic net (Enet) [10]. Later on, Li *et al.* proposed the network penalty (Network) [11], where $P_{\alpha }(\beta ^{m})=\frac {1}{2}(1-\alpha)(\beta ^{m})^{T}L\beta ^{m}+\alpha \|\beta ^{m}\|_{1}$, and *L* is the normalized Laplacian matrix for a network measuring the connections or the correlations of the variables. With this model, the connected variables in the network tend to be selected together. This penalty is further extended to $P_{\alpha }(\beta ^{m})=\frac {1}{2}(1-\alpha)(|\beta |^{m})^{T}L|\beta |^{m}+\alpha \|\beta ^{m}\|_{1}$ to tackle the case when the highly correlated variables have opposite contributions to the response (Abs-Network) [12, 13].

When we identify the biomarkers from multiple datasets generated for the same biological question, different biomarkers may be selected when we do the experiments in each individual dataset. This may be due to the batch effects from different platforms/experimental conditions, or the high co-linearity among the variables. In reality, the same variables should contribute to their corresponding response equally. Thus we assume *β*^1^=*β*^2^=⋯*β*^*M*^=*β*. To estimate these parameters, direct merging all the datasets together is one choice. However, it cannot explain the heterogeneity of different datasets. To explain the heterogeneity, and to make the final response appear with high probabilities, we set the constant term in the model to be different for different samples. We let $\beta _{0,i}^{m}$ be the constant term corresponding to the sample *i* in the *m*-th experiment. Due to the homogeneity within the same dataset, all the parameters should be the same for the same dataset. Thus we add constraints to make the model satisfy this condition. Our formulation now becomes:

3 $$\begin{array}{@{}rcl@{}} \min_{\beta_{0},\beta} &&\frac{1}{N}\sum_{m=1}^{M}\sum_{i=1}^{N^{m}} (-Y_{i}^{m}\cdot \left(\beta_{0,i}^{m}+X_{i}^{m}\beta\right)\\&&\qquad+\log\left(1+\exp\left(\beta_{0,i}^{m}+X_{i}^{m}\beta\right)\right)) \\&&\qquad+\lambda P_{\alpha}(\beta)+\mu \beta_{0}^{T}\tilde L\beta_{0}\\ & &=\frac{1}{N} \sum_{m=1}^{M}-\ell\left(\beta_{0}^{m},\beta\right)+\lambda P_{\alpha}(\beta)+\mu \beta_{0}^{T}\tilde L\beta_{0}. \end{array} $$

Here, $\beta _{0}=\left (\left (\beta _{0}^{1}\right)^{T},\left (\beta _{0}^{2}\right)^{T},\cdots,\left (\beta _{0}^{M}\right)^{T}\right)^{T}$, $\beta _{0}^{m}=\left (\beta _{0,1}^{m},\beta _{0,2}^{m},\cdots,\beta _{0,N^{m}}^{m}\right)^{T}, m =1,2,\cdots M$. *μ* is a parameter to control the importance of the penalty term $\beta _{0}^{T}\tilde L\beta _{0}$. $\tilde L$ is the Laplacian matrix for the sample relation network, where if two samples are from the same dataset, we assign an edge between them, otherwise, there is no edge. By minimizing the term $\beta _{0}^{T}\tilde L\beta _{0}$, $\beta _{0,i}^{m}$ will tend to be the same for different *i* and a fixed *m*, and it depends on *m* when sample *i* is from different experiments. This penalty helps make the constant term in logistic regression be the same for the same dataset, and allowing the differences across different datasets.

To solve the optimization problem (3), we notice that its formulation is similar to (2), except the constraint on *β*_0_. Thus we can apply similar methods to the one solving (2). In [9, 39], proximal Newton method is applied to solve the problem (2) [40]. This method mainly includes two steps: first a Newton step is applied to the log-likelihood term to get a temporal point; then the original optimization problem is approximated at this point by a quadratic function with the original penalty kept. Usually this quadratic optimization problem can be solved efficiently. Using the same technique, for our formulation (3), we first derive a temporal point with Newton method for the log-likelihood term. Different from (2), here *β*_0_ is a vector of size *N*. To take advantage of the standard logistic regression, we let $\tilde {\mathbf {X}}=(\mathbf {X},I_{N\times N})$, $\tilde {\beta }=\left (\beta ^{T},\beta _{0}^{T}\right)^{T}$, where *I*_*N*×*N*_ is an identity matrix. Then for any sample *i* in **X**, we have $\log \left (\frac {\mathbf {p}_{i}}{1-\mathbf {p}_{i}}\right)=\tilde {\mathbf {X}}_{i}\tilde {\beta },$ where **p**_*i*_=*P*(**Y**_*i*_=1|**X**_*i*_),*i*=1,2,⋯,*N* for the integrated datasets.

Now we can use standard Newton method to get a new point $\tilde {\beta }^{temp}$ by computing:

4 $$\begin{array}{@{}rcl@{}} \hspace{21pt} Z=\tilde{\mathbf{X}}\tilde\beta^{\text{old}}+{\mathbf{W}}^{-1}(\mathbf{Y}-\mathbf{p}), \end{array} $$

$$\begin{array}{@{}rcl@{}}\tilde\beta^{temp}=\left(\tilde{\mathbf{X}}^{T}{\mathbf W}\tilde{\mathbf{X}}\right)^{-1}\tilde{\mathbf{X}}^{T}\mathbf{W}Z. \end{array} $$

Here **p**=(**p**_1_,**p**_2_,⋯,**p**_*N*_)^*T*^, **W**=*diag*(**p**)·*diag*(**1−p**). $\left (\tilde {\mathbf {X}}^{T}\mathbf {W}\tilde {\mathbf {X}}\right)$ is the Hessian matrix for the likelihood function.

The quadratic approximation problem to be solved is:

5 $${}\text{prox}_{H}\left(\tilde\beta^{temp}\right)\!\,=\, \!\arg\min_{\tilde\beta} \frac{1}{2}\|\tilde\beta^{temp} \,-\,\tilde\beta\|_{H}^{2}+\lambda P_{\alpha}(\beta)+\mu \beta_{0}^{T}\tilde L\beta_{0}, $$

where $H=\tilde {\mathbf {X}}^{T}{\mathbf W}\tilde {\mathbf {X}}$. It is equivalent to the following problem:

6 $$\begin{array}{@{}rcl@{}} \text{prox}_{H}\left(\tilde\beta^{temp}\right) &\,=\,&\arg\min_{\tilde\beta} \frac{1}{2N}\!\!\sum\limits_{i=1}^{N}\mathbf{p}_{i}(1\,-\,\mathbf{p}_{i})(Z_{i}\,-\,\beta_{0,i}\,-\,\mathbf{X}_{i}\beta)^{2}\\ &&+\lambda P_{\alpha}(\beta)+\mu \beta_{0}^{T}\tilde L\beta_{0}. \end{array} $$

To solve problem (6), we refer to the coordinate descent algorithm [41]. Given *β*, the objective function is convex and smooth with respect to *β*_0_, thus we can compute the elements in *β*_0_ simultaneously. Given *β*_0_, the objective function is nonsmooth when *L*_1_ penalty exists, and may be convex or not depending on the penalty term for *β*. When we use the Abs-Network penalty, it is nonconvex. However, as described in [41, 42], this term follows the regularity condition, which implies that if moving along all coordinate directions fail to decrease the objective function, it arrives at the local minimum. Thus we can use the coordinate descent algorithm. In the following, we derive the computation procedure for (6) with Abs-Network penalty. For other penalties mentioned above, the computation procedure is similar. Given *β*, *β*_0_ should satisfy the equation:

7 $$\begin{array}{@{}rcl@{}} \left(\frac{1}{N}\mathbf{W}+2\mu\tilde L\right)\beta_{0}=\mathbf{W}(Z-\mathbf{X}\beta), \end{array} $$

which is obtained by computing the partial derivative with respect to *β*_0_. Given *β*_0_, we compute *β* using cyclic coordinate descent, which can be computed using:

8 $$\begin{array}{@{}rcl@{}}\beta_{k}=S(u_{k},v_{k}),\end{array} $$

where

$$u_{k}=\frac{\frac{1}{N}\sum_{i=1}^{N}\mathbf{W}_{i,i}\mathbf{X}_{i,k}\left(Z_{i}-\hat{\beta}_{0,i}-\sum_{j\neq k}\mathbf{X}_{ij}\hat{\beta}_{j}\right)}{\frac{1}{N}\sum_{i=1}^{N} \mathbf{W}_{i,i}\mathbf{X}_{i,k}^{2}+\lambda(1-\alpha)\sum_{j\neq k}\frac{A_{k,j}}{d_{k}}},$$

$$v_{k}=\frac{\lambda\alpha-\lambda(1-\alpha)\sum_{j\neq k}\frac{|\hat\beta_{j}|}{\sqrt{d_{k}}\sqrt{d_{j}}}A_{k,j}}{\frac{1}{N}\sum_{i=1}^{N} \mathbf{W}_{i,i}\mathbf{X}_{i,k}^{2}+\lambda(1-\alpha)\sum_{j\neq k}\frac{A_{k,j}}{d_{k}}}. $$

Here, we use $\hat \beta _{0}, \hat \beta _{j}$ to denote the fixed parameters in the coordinate descent process. *S*(*u*,*v*) is a soft-thresholding function defined as: *S*(*u*,*v*)=*sign*(*u*) max(|*u*|−*v*,0). *A*_*k*,*j*_ denotes the (*k*,*j*) entry in the network adjacency matrix for the variables, *d*_*j*_ denotes the degree of the *j*-th variable in the network of variables. Algorithm 1 shows the full process of computing $\tilde \beta $. For other penalties, we can infer the algorithm similarly.


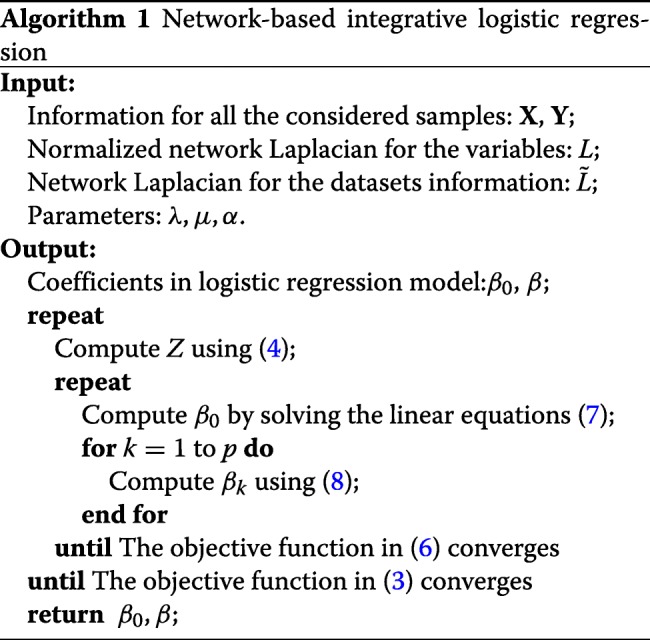


After the computation, we can get *β* and *β*_0_. Then we average the value $\beta _{0}^{m}$ to get an estimate of the constant term for the data in the *m*-th experiment, and do the prediction.

## Results

In this section, we first evaluate the proposed integrative logistic regression model using simulation studies, we then apply the method to multiple gene expression datasets for studying breast cancer metastasis.

### Simulation study

The experiments are designed to classify the case/control samples using gene expression datasets. We simulated the gene expression datasets using the similar method as that in [11]. Suppose we have *n*_*TF*_ transcription factors (TFs) and each regulates *n*_*RG*_ genes. The resulting regulatory network includes *n*_*TF*_+*n*_*RG*_ genes and the edges between each of the TFs and the regulated genes. We assume four TFs and the genes that they regulate are related to the response *Y*. We generated the input variables using the following distributions:

- The expression levels for the *n*_*TF*_ TFs follow standard normal $X_{TF_{j}}\sim N(0, 1)$;
- The expression levels of the TF and the gene that it regulates are jointly distributed as a bivariate normal with a correlation of 0.7, which implies that conditioning on the expression level of the TF, the regulated gene expression level follows normal distribution: $N(0.7X_{TF_{j}}, 0.51)$.

We designed two settings of the regression coefficients. The first one is shown in (9),

9 $$\begin{array}{@{}rcl@{}} \beta&\,=\,&\left[\sqrt{5},\underbrace{\frac{5}{\sqrt{10}},\cdots,\frac{5}{\sqrt{10}}}_{7},\frac{-5}{\sqrt{10}},\frac{-5}{\sqrt{10}},\frac{-5}{\sqrt{10}}, \right.\\ &&-\sqrt{5},\underbrace{\frac{-5}{\sqrt{10}},\cdots,\frac{-5}{\sqrt{10}}}_{7},\frac{5}{\sqrt{10}},\frac{5}{\sqrt{10}},\frac{5}{\sqrt{10}},\\ &&\sqrt{3},\underbrace{\frac{3}{\sqrt{10}},\cdots,\frac{3}{\sqrt{10}}}_{7},\frac{-3}{\sqrt{10}},\frac{-3}{\sqrt{10}},\frac{-3}{\sqrt{10}},\\ &&\left.-\sqrt{3},\underbrace{\frac{-3}{\sqrt{10}},\cdots,\frac{-3}{\sqrt{10}}}_{7},\frac{3}{\sqrt{10}},\frac{3}{\sqrt{10}},\frac{3}{\sqrt{10}},0,\cdots,0\right]. \end{array} $$

The constant term *β*_0_ is set to be different in multiple datasets. Here, we generated four different datasets, and the mean of $\beta _{0}^{m}$: $\bar \beta _{0}^{m} $ for *m*=1,2,3,4 is set to be − 3, − 1, 1, and 3. $\beta _{0,i}^{m}$ for each sample *i* follows $N\left (\bar \beta _{0}^{m},0.5\right)$. In this case, when integrating the four datasets, one main concern for predicting *Y* is the batch effects shown in *β*_0_. *Y*_*i*_ is generated following Bernoulli distribution with the probability *P*(*Y*_*i*_=1|*X*_*i*_).

The second setting is that the regression coefficients *β* for the TFs and their regulated genes are generated using uniform distribution in [0,3], with their signs shown in the following vector:

10 $$\begin{array}{*{20}l} {}sign(\beta)&=\left[\underbrace{1,\cdots,1}_{11},\underbrace{-1,\cdots,-1}_{11}, \underbrace{1,\cdots,1}_{7},\underbrace{-1,\cdots,-1}_{4}, \right.\\ &\left.\quad\underbrace{-1,\cdots,-1}_{7},\underbrace{1,\cdots,1}_{4}\right], \end{array} $$

where ‘1’ means the corresponding coefficient is positive, and ‘ − 1’ negative. Similarly, we set the mean of $\beta _{0}^{m}$ for *m*=1,2,3,4 to be − 3,−1,1, and 3. The heterogeneity of the datasets is shown in both the regression coefficients and the constant term for different datasets. *Y*_*i*_ is also generated following Bernoulli distribution with the probability *P*(*Y*_*i*_=1|*X*_*i*_).

For each setting, we generated 100 training samples and 100 test samples for the four datasets. We set *μ*=1 and *α*=0.5 directly except the LASSO penalty, and used 5-fold cross validation (CV) to train the model and got the parameter *λ*. Then we applied the model obtained using the full training set with the parameters that gave the best AUC (area under ROC curve) to see the prediction results in the test set. We took prediction sensitivity, specificity, accuracy, AUC, and the variable selection precision, recall, *F*_1_ score to measure the prediction and variable selection results of the model. The variable penalty term is set to be LASSO [2], elastic net (Enet) [10], network constraint (Network) [11], and network-regularized penalty using absolute value of the coefficients (Abs-Network) [12]. We note that better results are expected if we use CV to choose all these three parameters (*μ*,*α*,*λ*) together at the cost of more parameter tuning computation time.

To evaluate the performance of the proposed method, we compared it with the methods without integration, direct data merging, and MetaLasso [36]. For the results obtained without integration, we trained the model in each of the four training datasets separately, and predicted the samples in all the four test sets. We then recorded the best result among the four. For direct data merging, we merged the four training sets as the training set, and the four test sets as the test set, followed by the penalized logistic regression. We implemented the R package ‘MetaLasso’ for MetaLasso [36]. We added the prefix ‘Int-’ to denote the method after integration using the corresponding penalty, and ‘Merge-’ to denote a direct merging of all the datasets. We implemented the whole computation process for 30 times for each method, and computed the mean and standard deviation (sd) values of the seven evaluation measures.

Tables 1 and 2 show the prediction and variable selection results for setting 1, and Tables 3 and 4 show the results for setting 2. We highlighted the highest values for each measure. For the prediction results, it is clear that adding network constraints to integrate multiple datasets in the model improves the results under both of our simulation settings. Normally, direct data merging outperforms the methods without integration, and integration outperforms direct merging. For MetaLasso, it only uses the LASSO penalty, and outperforms LASSO and Merge-LASSO. But it is not as good as our proposed integration method using LASSO penalty. This shows that our integration technique can capture more information in multiple datasets. For the variable selection results, the highest *F*_1_ score is achieved using Int-Abs-Network, though Merge-Network and Merge-Abs-Network achieve the highest precision. This should come from the fact that data merging enhances the signal of the important variables and variable network constraints combine the related variables. However, merging may miss some associated genes due to the heterogeneity across multiple datasets, which may lead to a lower recall in some experiments. Compared to it, Int-Abs-Network performs more robust and gets the highest *F*_1_ score.

**Table 1:** Prediction results for simulation setting 1

	Prediction			
Method	Sensitivity	Specificity	Accuracy	AUC
LASSO	0.63(0.05)	0.62(0.04)	0.62(0.02)	0.66(0.02)
Enet	0.65(0.05)	0.64(0.05)	0.63(0.02)	0.68(0.02)
Network	0.82(0.06)	0.82(0.06)	0.81(0.05)	0.89(0.05)
Abs-Network	0.82(0.05)	0.82(0.06)	0.81(0.04)	0.89(0.04)
Merge-LASSO	0.65(0.04)	0.65(0.06)	0.63(0.02)	0.68(0.02)
Merge-Enet	0.65(0.05)	0.64(0.05)	0.63(0.02)	0.68(0.02)
Merge-Network	0.87(0.04)	0.88(0.03)	0.88(0.03)	0.95(0.02)
Merge-Abs-Network	0.88(0.04)	0.88(0.03)	0.88(0.02)	0.95(0.02)
Int-LASSO	0.88(0.02)	0.88(0.02)	0.88(0.02)	0.96(0.01)
Int-Enet	0.88(0.02)	0.88(0.02)	0.88(0.02)	0.96(0.01)
Int-Network	0.89(0.02)	**0.90(0.02)**	0.89(0.01)	0.96(0.01)
Int-Abs-Network	**0.90(0.02)**	**0.90(0.02)**	**0.90(0.01)**	**0.97(0.01)**
MetaLasso	0.75(0.05)	0.76(0.04)	0.76(0.04)	0.84(0.04)

*β* is shown in (9), $\left (\bar \beta _{0}^{1},\bar \beta _{0}^{2},\bar \beta _{0}^{3},\bar \beta _{0}^{4}\right)=(-3,-1,1,3)$

The maximum value for each measure is highlighted using boldface font

**Table 2:** Variable selection results for simulation setting 1

	Variable selection		
Method	Precision	Recall	*F*_1_ Score
LASSO	0.93(0.02)	0.26(0.06)	0.60(0.06)
Enet	0.90(0.04)	0.41(0.06)	0.61(0.06)
Network	0.85(0.02)	0.91(0.05)	0.80(0.06)
Abs-Network	0.82(0.02)	0.95(0.05)	0.81(0.06)
Merge-LASSO	0.94(0.02)	0.49(0.05)	0.62(0.05)
Merge-Enet	0.94(0.02)	0.56(0.04)	0.61(0.07)
Merge-Network	**0.99(0.01)**	0.94(0.03)	0.87(0.03)
Merge-Abs-Network	**0.99(0.01)**	**0.98(0.02)**	0.88(0.03)
Int-LASSO	0.95(0.01)	0.49(0.05)	0.88(0.02)
Int-Enet	0.96(0.01)	0.65(0.04)	0.88(0.02)
Int-Network	0.94(0.04)	0.96(0.03)	0.89(0.01)
Int-Abs-Network	0.91(0.05)	**0.98(0.02)**	**0.90(0.01)**
MetaLasso	0.94(0.01)	0.05(0.02)	0.75(0.04)

*β* is shown in (9), $\left (\bar \beta _{0}^{1},\bar \beta _{0}^{2},\bar \beta _{0}^{3},\bar \beta _{0}^{4}\right)=(-3,-1,1,3)$

The maximum value for each measure is highlighted using boldface font

**Table 3:** Prediction results for simulation setting 2

	Prediction			
Method	Sensitivity	Specificity	Accuracy	AUC
LASSO	0.63(0.05)	0.64(0.08)	0.62(0.02)	0.66(0.03)
Enet	0.61(0.04)	0.63(0.06)	0.61(0.03)	0.65(0.03)
Network	0.83(0.04)	0.85(0.06)	0.84(0.04)	0.92(0.04)
Abs-Network	0.85(0.05)	0.84(0.05)	0.84(0.03)	0.92(0.03)
Merge-LASSO	0.63(0.05)	0.63(0.06)	0.61(0.02)	0.66(0.02)
Merge-Enet	0.62(0.04)	0.63(0.07)	0.61(0.02)	0.66(0.02)
Merge-Network	0.82(0.04)	**0.87(0.03)**	0.84(0.03)	0.93(0.02)
Merge-Abs-Network	0.81(0.04)	0.86(0.04)	0.83(0.03)	0.92(0.03)
Int-LASSO	0.82(0.03)	0.89(0.03)	0.85(0.03)	0.93(0.02)
Int-Enet	0.82(0.04)	0.89(0.03)	0.85(0.03)	0.94(0.02)
Int-Network	0.88(0.04)	**0.87(0.03)**	0.87(0.02)	**0.95(0.02)**
Int-Abs-Network	**0.89(0.04)**	0.87(0.04)	**0.88(0.02)**	**0.95(0.02)**
MetaLasso	0.81(0.03)	0.82(0.04)	0.81(0.04)	0.90(0.03)

The sign of *β* is shown in (10), $\left (\bar \beta _{0}^{1},\bar \beta _{0}^{2},\bar \beta _{0}^{3},\bar \beta _{0}^{4}\right)=(-3,-1,1,3)$

The maximum value for each measure is highlighted using boldface font

**Table 4:** Variable selection results for simulation setting 2

	Variable selection		
Method	Precision	Recall	*F*_1_ Score
LASSO	0.91(0.04)	0.28(0.06)	0.60(0.07)
Enet	0.91(0.04)	0.35(0.07)	0.62(0.06)
Network	0.85(0.03)	0.74(0.12)	0.84(0.05)
Abs-Network	0.83(0.03)	0.77(0.13)	0.84(0.03)
Merge-LASSO	0.95(0.01)	0.42(0.08)	0.60(0.06)
Merge-Enet	0.95(0.01)	0.50(0.07)	0.61(0.05)
Merge-Network	**0.98(0.01)**	0.74(0.08)	0.84(0.03)
Merge-Abs-Network	**0.98(0.01)**	0.77(0.08)	0.84(0.03)
Int-LASSO	0.95(0.01)	0.43(0.09)	0.85(0.02)
Int-Enet	0.96(0.01)	0.64(0.07)	0.86(0.03)
Int-Network	0.93(0.03)	0.83(0.07)	0.87(0.03)
Int-Abs-Network	0.92(0.04)	**0.84(0.09)**	**0.88(0.03)**
MetaLasso	0.94(0.01)	0.04(0.02)	0.81(0.04)

The sign of *β* is shown in (10), $\left (\bar \beta _{0}^{1},\bar \beta _{0}^{2},\bar \beta _{0}^{3},\bar \beta _{0}^{4}\right)=(-3,-1,1,3)$

The maximum value for each measure is highlighted using boldface font

### Real data study

We downloaded two datasets GSE2034 and GSE1456 from Gene Expression Omnibus (GEO). These two datasets were generated for studying breast cancer metastasis. The information of the samples is shown in Table 5. Both datasets were measured on Affymetrix HGU133 microarrays, and each dataset includes 22283 transcripts. We combined those probes corresponding to the same gene using their mean value as the gene expression level. We then downloaded the protein-protein interaction (PPI) data from https://thebiogrid.org for the humans, and removed the genes that have no information in the PPI network. We chose 2000 genes with the largest variance from each dataset and took their intersection as our considered gene set. Finally a total of 1456 genes were selected. For each gene, we imputed the missing value using the mean value of the gene, and normalized the expression of each gene.

**Table 5:** Datasets summary [14]

Dataset	Publication	# Patients	Classification	# patients
GSE2034	[44]	242	time to relapse ≤ 5y & relapse=True	95
			time to relapse > 7y & relapse=False	147
GSE1456	[45]	111	time to relapse ≤ 5y & relapse=True	35
			time to relapse > 7y & relapse=False	76

We applied three types of methods to these two datasets. The first type of method applied logistic regression model with the four penalties to the merged data directly. The second type is MetaLasso, and our proposed method is as the third type. When using our method, we set *μ*=1 and *α*=0.5 except LASSO penalty. We selected the parameter *λ* using 3-fold cross validation between 0.02 to 0.1 with a step size 0.02 and got the AUC under ROC curve. We then trained the model using the whole dataset and got the biomarkers. Table 6 shows the mean of AUCs and its standard deviation when doing CV. It’s clear that our method outperforms direct data merging. MetaLasso achieved the AUC value 0.62 with sd 0.02, and it selected three genes as the biomarkers. In [14], several biomarker identification methods have been applied to these two datasets separately. The best AUC for GSE2034 is 0.690 and 0.736 for GSE1456, respectively. The stability for the selected genes measured using Jaccard index is about 0.2, which means the intersection of the selected genes in both datasets over their union is about 0.2. This shows the instability of biomarker identification from different datasets. With our method, using the same biomarkers, we can get a comparable AUC 0.70. We note that, if we tune all the three parameters *λ*,*α*,*μ* together, we may get better results at the cost of more computational time.

**Table 6:** Real data results. MetaLasso achieved the AUC 0.62(0.02), and selected 3 genes as biomarkers

	Data Merging		Our model	
Penalty	AUC	# Genes	AUC	# Genes
LASSO	0.67(0.01)	59	0.70(0.03)	122
Enet	0.67(0.01)	306	0.69(0.02)	104
Network	0.58(0.01)	255	0.70(0.04)	214
Abs-Network	0.59(0.03)	285	0.67(0.01)	270

As we know, when LASSO penalty is applied, less genes will be selected, while when the elastic penalty is added, the correlated genes can be selected. By adding the PPI network related penalties, we aim at finding the subnetworks that cooperatively contribute to the disease development. Table 6 also shows the number of the selected genes for different penalties. Since the development of disease is a very complex process, subnetwork biomarkers are reasonable. We presented the subnetwork biomarker identification using Abs-Network penalty in the following.

**Fig. 1:**
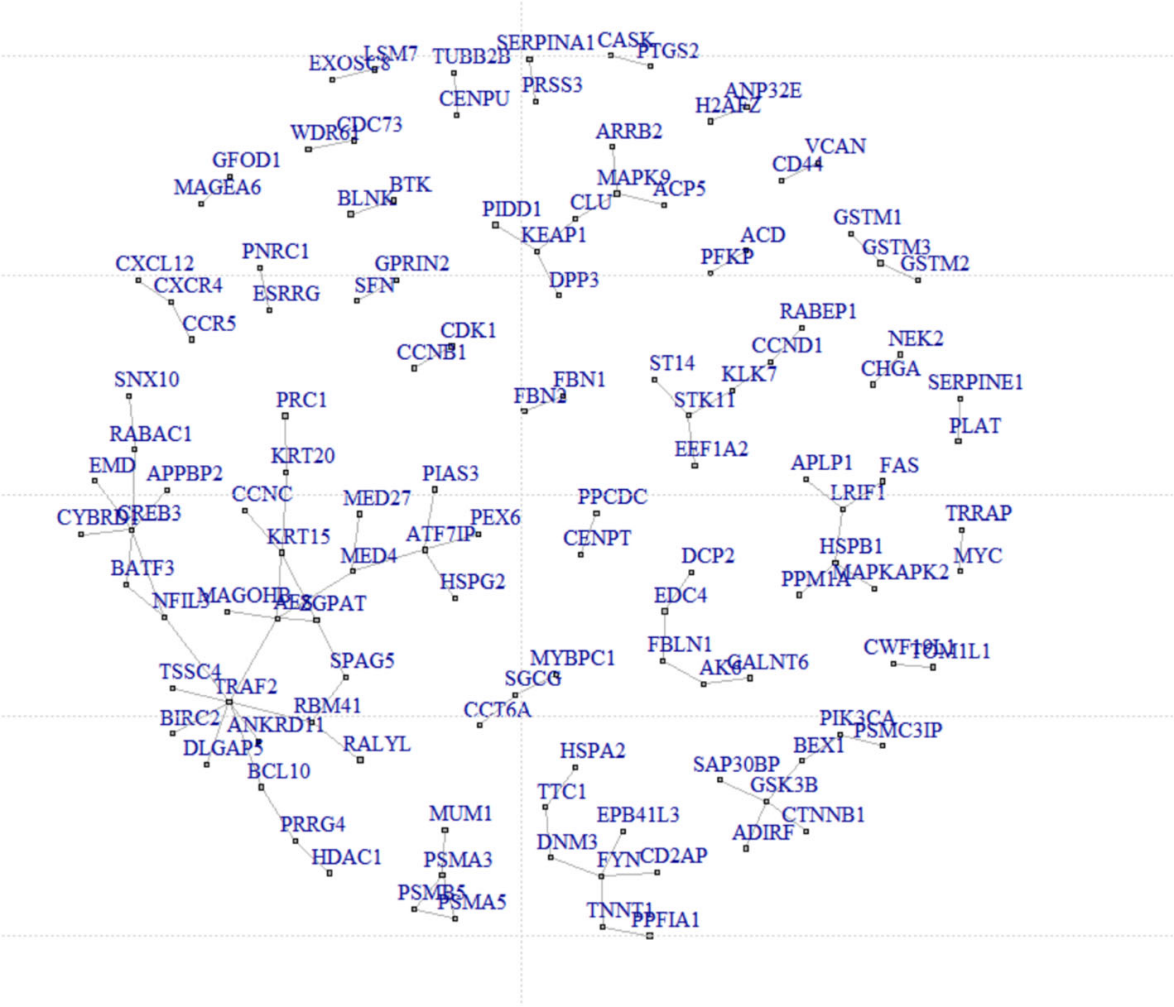
Identified subnetwork biomarkers using network-based integrative logistic regression with Abs-Network penalty

**Fig. 2:**
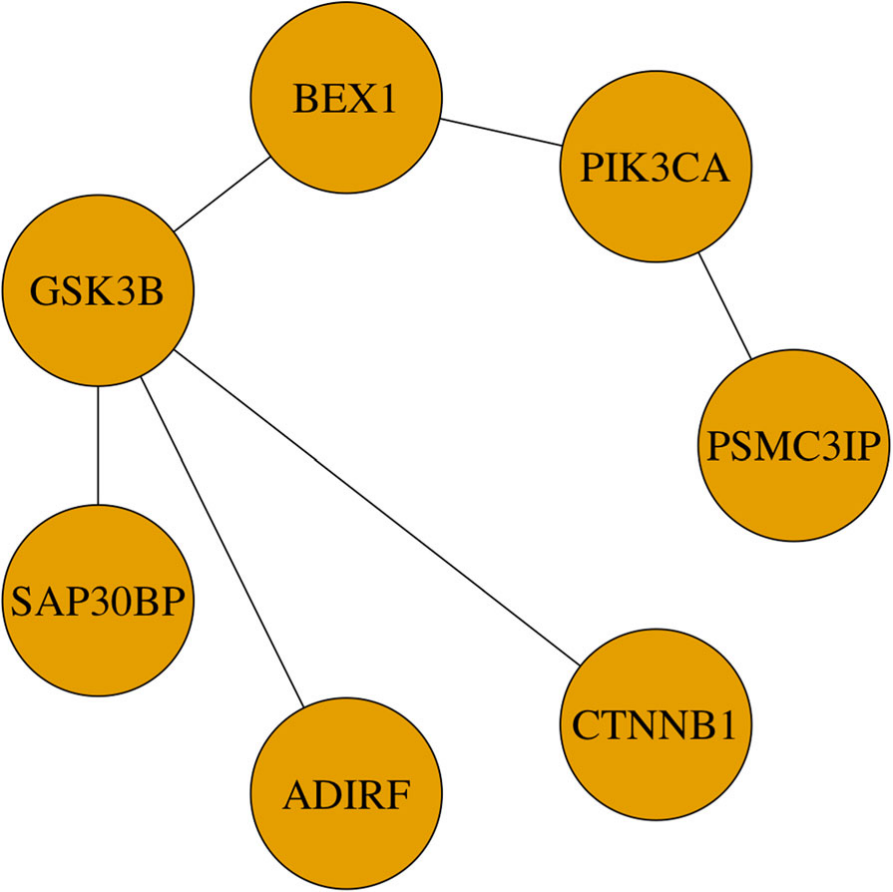
One identified subnetwork biomarker using network-based integrative logistic regression with Abs-Network penalty

When using Abs-Network penalty, 270 genes were selected, among which there are 30 connected subnetworks. We put all the 30 subnetworks in Additional file 1. Figure 1 shows the connected components. We also did gene ontology (GO) enrichment analysis and KEGG pathway enrichment analysis for these subnetworks using “clusterProfiler” [43]. Twenty one of all these networks are enriched by GO: CC, MF and BP, and KEGG pathway. We put all these enrichment results in Additional files 2, 3, 4, and 5. One typical subnetwork is shown in Fig. 2. This subnetwork is enriched by KEGG pathway hsa05224: breast cancer with an adjusted *p*-value 10^−4^. There are 7 genes in this subnetwork, of which three genes (“GSK3B”, “CTNNB1”, “PIK3CA”) are associated with breast cancer. And these three genes are also associated with some other cancers such as endometrial cancer, colorectal cancer, prostate cancer, and others. GSK3B interacts with CTNN1, while it interacts with PIK3CA through BEX1.

## Discussion

Biomarker identification has been a hot research topic for several years. Many mathematical and statistical models and algorithms have been proposed to tackle this problem. However, due to the heterogeneity of datasets from different platforms or laboratories, the biomarkers inferred from one single dataset are lack of reproducibilities. Even different datasets are generated for the same purpose, the intersection of the inferred biomarkers is small. This motivates us to consider the integrative methods for robust biomarker identification using multiple datasets.

In this work, we assumed the regression coefficients of the variables for multiple datasets are the same. The differences in predicting the response of each sample lie in the constant term. To combine the information from different samples, we added the network penalty to make the constant term within the same dataset be the same. To achieve the biomarker identification purpose, we added more penalties than LASSO, such as network related penalties to select the subnetworks as biomarkers. We then developed proximal Newton method to solve the optimization problem, and gave the detailed formulations for the Abs-Network penalty. Algorithms for other formulations can be easily inferred. Since this algorithm involves solving linear equations, it is slower than that solving the model without integration term. Developing faster algorithms so as to apply the model to larger dataset is very important.

We applied the proposed model to both simulated datasets and real datasets. For the simulation study, it is not easy to make simulations similar to the real data. We tried two different settings to see the performance of the model. Both experiments gave reasonable results. In the real data study, we integrated two breast cancer gene expression datasets. We compared the results with direct merging the datasets and MetaLasso, and we checked the existing works on biomarker identification and prediction in each dataset separately. Our method performs much better than direct merging and MetaLasso. And it achieved results comparable to the best results in each single dataset. All these results show the good performance of our proposed method. In our model, we assumed the sources of the test dataset are included in that of the training dataset, thus when we do prediction, we can directly use the corresponding constant term in logistic regression. This limits the application of the proposed model for the datasets whose sources are not known.

In this study, we tested our method in only one real data setting. Other datasets may not have the same properties as our tested datasets. Thus applying our model to more real datasets, and incorporating more information to the model so as to improve the prediction accuracy is one of the future works.

## Conclusions

In this work, we proposed an integrative method for classification based on logistic regression model. By adding a network-based penalty for the constant term in logistic regression for the samples from different datasets, both the homogeneity within each dataset and the heterogeneity between different datasets are kept. After adding network related penalties besides LASSO, subnetwork biomarkers can be identified. In both simulation datasets and the real datasets, the proposed method shows good performance. This method may help better identify the biomarkers by integrating multiple datasets.

## Additional files

Additional file 1 The identified subnetwork biomarkers. The identified subnetwork biomarkers using network-based integrative logistic regression with Abs-Network penalty. (TXT 2 kb)

Additional file 2 GO:BP enrichment results of the subnetwork biomarkers. The enrichment of GO: BP is included. (XLSX 220 kb)

Additional file 3 GO:CC enrichment results of the subnetwork biomarkers. The enrichment of GO: CC is included. (XLSX 46 kb)

Additional file 4 GO:MF enrichment results of the subnetwork biomarkers. The enrichment of GO: MF is included. (XLSX 69 kb)

Additional file 5 KEGG pathway enrichment results of the subnetwork biomarkers. The enrichment of KEGG pathway is included. (XLSX 36 kb)

## Abbreviations

Abs-Network: Network regularized penalty using absolute value of the coefficients
Enet: Elastic net
LASSO: Least absolute shrinkage and selection operator
